# How Much They Eat in New-York

**Published:** 1854-12

**Authors:** 


How much they Eat in New-York.—During the three months ending the 1st inst., two hundred and nineteen thousand six hundred and thirteen animals were slaughtered for food in New-York City. During the nine months of the year, the total number of animals offered up on the altar of appetite in that city, reached seven hundred thousand seven hundred and fourteen, or, at the rate of seventeen thousand nine hundred and sixty-six per week, or nearly one million per year! The population is about five hundred thousand—that is an average of two animals per year to each inhabitant. Not much starving in New-York, at this rate.—*Albany Register.*

